# Two Family B DNA Polymerases From *Aeropyrum pernix*, Based on Revised Translational Frames

**DOI:** 10.3389/fmolb.2018.00037

**Published:** 2018-04-16

**Authors:** Katsuya Daimon, Sonoko Ishino, Namiko Imai, Sachiyo Nagumo, Takeshi Yamagami, Hiroaki Matsukawa, Yoshizumi Ishino

**Affiliations:** Graduate School of Bioresource and Bioenvironmental Sciences, Kyushu University, Fukuoka, Japan

**Keywords:** Archaea, DNA replication, DNA polymerase, heat-stable enzyme, PCR

## Abstract

Living organisms are divided into three domains, Bacteria, Eukarya, and Archaea. Comparative studies in the three domains have provided useful information to understand the evolution of the DNA replication machinery. DNA polymerase is the central enzyme of DNA replication. The presence of multiple family B DNA polymerases is unique in Crenarchaeota, as compared with other archaeal phyla, which have a single enzyme each for family B (PolB) and family D (PolD). We analyzed PolB1 and PolB3 in the hyperthermophilic crenarchaeon, *Aeropyrum pernix*, and found that they are larger proteins than those predicted from the coding regions in our previous study and from public database annotations. The recombinant larger PolBs exhibited the same DNA polymerase activities as previously reported. However, the larger PolB3 showed remarkably higher thermostability, which made this enzyme applicable to PCR. In addition, the high tolerance to salt and heparin suggests that PolB3 will be useful for amplification from the samples with contaminants, and therefore it has a great potential for diagnostic use in the medical and environmental field.

## Introduction

DNA polymerase catalyzes phosphodiester bond formation between the terminal 3′-OH of the primer and the α-phosphate of the incoming triphosphate to extend the DNA strand based on the template DNA sequence. Numerous DNA polymerases have been reported since *Escherichia coli* DNA polymerase I was first identified in 1956 (Bessman et al., 1956). DNA polymerases are now classified into seven families, A, B, C, D, E, X, and Y, based on the amino acid sequence similarity (Braithwaite and Ito, 1993; Cann and Ishino, 1999; Ohmori et al., 2001; Lipps et al., 2003). For example, *E. coli* has five DNA polymerases. Pol I, II, and III belong to families A, B, and C, respectively, and Pol IV and Pol V are classified in family Y, which mostly includes the DNA polymerases for translesion synthesis (TLS). The bacterial PolIII and the eukaryal Pols α, δ, and ε, the essential replicases for the DNA replication process, are family C and family B enzymes, respectively. The eukaryotic enzymes for TLS, including Pol η, *l*, and κ, belong to family Y, as do those in the bacterial domain. The fundamental ability to incorporate a deoxymononucleotide into the DNA strand is generally conserved among the DNA polymerases. However, the specific properties, such as processivity, fidelity, and substrate nucleotide selectivity, vary depending on the families (Ishino and Ishino, 2013).

The distribution and functional sharing of DNA polymerases in Archaea, the third domain of life, have been analyzed since the early 1990s. It was exciting to find a gene encoding a eukaryotic -like family B DNA polymerase in Archaea (Perler et al., 1992; Pisani et al., 1992; Uemori et al., 1993), and furthermore, two different family B DNA polymerases were identified in the archaeal genome of *Pyrodictium occultum* (Uemori et al., 1995). These findings, reported before the total genome sequencing, provided strong motivation to study DNA replication in Archaea, because the DNA replication system consisting of multiple family B DNA polymerases may be conserved between Archaea and Eukarya. However, some archaea have only one family B DNA polymerase, and as an alternative, their genomes encode an archaea-specific DNA polymerase, which has never been found in Bacteria and Eukarya (Uemori et al., 1997; Ishino et al., 1998). This DNA polymerase, originally discovered in *Pyrococcus furiosus*, consists of two proteins, DP1 and DP2, and their deduced amino acid sequences are not conserved in the DNA polymerase families. Therefore, this DNA polymerase was proposed to be PolD, from the family D (Cann and Ishino, 1999). PolD has now been found in the genomes of all of the archaeal phyla, except for Crenarchaeota (Ishino and Ishino, 2014). Based on our current knowledge about the distribution of DNA polymerases in Archaea, one PolB and one PolD are present in most archaeal cells, but two PolBs, without PolD are probably working in crenarchaeal cells. Therefore, one of the questions to be clarified in archaeal DNA replication is how the two PolBs share the functions in Crenarchaeota.

After our finding of two family B DNA polymerases in *P. occultum*, as described above (Uemori et al., 1995), we also found two DNA polymerase activities in the cell extracts of *Aeropyrum pernix*, another hyperthermophilic crenarchaeon, and their gene sequences revealed that both of these DNA polymerases belong to family B (Cann et al., 1999). The remarkable difference was their aphidicolin sensitivity and heat stability *in vitro*. Interestingly, a third gene encoding a family B DNA polymerase was found in the *Sulfolobus solfataricus* genome, and the three enzymes were designated as PolB1, B2, and B3 (Edgell et al., 1997). Only PolB1 and PolB3 contain the conserved exonuclease and polymerase motifs (Rogozin et al., 2008). The two enzymes, designated as PolI and PolII from *P. occultum* and *A. pernix* in our previous reports, were PolB1 and PolB3, respectively, and the euryarchaeal single PolBs belong to PolB3 (Rogozin et al., 2008).

The functions of these family B DNA polymerases in the crenarchaeal cells have not been elucidated, and thus further studies are required. In this study, we analyzed PolB1 and PolB3 in *A. pernix* cells, and found that both enzymes are produced as longer peptide chains than those predicted from their coding regions shown in our previous report. We prepared the longer PolB1 and PolB3 as recombinant proteins, and characterized them *in vitro*.

## Materials and methods

### Sequence analysis

The *A. pernix* genome DNA sequence is available in GenBank (accession number: BA000002). Homologs of PolB1 and PolB3 were retrieved from the reference sequence database at the National Center for Biotechnology Information (NCBI), using BlastP with *A. pernix* PolB1 (APE0099) and PolB3 (APE2098.1) as queries. Among the 71 complete genome sequences of crenarchaeal organisms, we searched for possible initiation sites (GTG or TTG) in the nucleotide sequences upstream of the annotated ORFs of PolB1 and PolB3 in 24 species. Multiple alignments were performed with the MAFFT online service (Katoh et al., 2017), and the conserved motifs were manually identified.

### Cloning of the DNA polymerase genes from *A. pernix*

The five genes were amplified by PCR directly from *A. pernix* genomic DNA, using the primer sets PolB1LF/PolB1R, PolB1MF/PolB1R, PolB1SF/PolB1R, PolB3LF/PolB3R, and PolB3SF/PolB3R for PolB1L, PolB1M, PolB1S, PolB3L, and PolB3S, respectively (Supplementary Table 1). *P. furiosus* DNA polymerase B (PfuPolB) was prepared as described previously (Komori and Ishino, 2000). Each fragment amplified by PfuPolB was digested by NdeI and NotI (New England Biolabs), and ligated into the corresponding sites of the pET-21a(+) expression vector (Novagen) by T4 DNA ligase (New England Biolabs), and the inserted sequences were confirmed.

### Preparation of the recombinant proteins

Each recombinant protein was produced in *E. coli* BL21-CodonPlus (DE3)-RIL cells (Agilent). The transformed *E. coli* cells were grown in LB medium, containing 50 μg/ml ampicillin and 34 μg/ml chloramphenicol, at 37°C until the culture attained an OD_600_ of 0.5. Isopropyl β-D-thiogalactopyranoside was then added to a final concentration of 0.5 mM, and the cells were further grown for 18 h at 25°C. The cells were harvested and disrupted by sonication in buffer A (50 mM Tris-HCl, pH 8.0, 0.5 mM DTT, 0.1 mM EDTA, and 10% glycerol) containing 0.5 M NaCl. The crude protein samples, containing each overproduced DNA polymerase, were subjected to a western blot analysis. For further purification of ApePolB1L and ApePolB1S, the soluble cell extract was heated at 60°C for 20 min. The heat-resistant fraction was treated with 0.15% polyethyleneimine to remove the nucleic acids. The soluble proteins were precipitated by 80%-saturated ammonium sulfate. The precipitate was resuspended in buffer A containing 1.4 M (NH_4_)_2_SO_4_ and subjected to chromatography on a HiTrap Phenyl HP column (GE Healthcare), which was developed with a 1.4–0 M (NH_4_)_2_SO_4_ gradient in buffer A. The fraction containing ApePolB1 was dialyzed against buffer B (50 mM Tris-HCl, pH 8.6, 0.5 mM DTT, 0.1 mM EDTA, and 10% glycerol) containing 0.1 M NaCl. The dialysate was loaded onto a HiTrap Q HP column (GE Healthcare), which was developed with a 0.1–1 M NaCl gradient in buffer B. For the purification of ApePolB3L and ApePolB3M, the soluble cell extract was heated at 75°C for 20 min. The heat-resistant fraction was then treated with 0.15% polyethyleneimine. The soluble proteins were precipitated by 80%-saturated ammonium sulfate. The precipitate was dialyzed against buffer A containing 0.1 M NaCl and applied to a HiTrap SP HP column (GE Healthcare), which was developed with a 0–1 M NaCl gradient in buffer A. The protein concentrations were calculated by measuring the absorbance at 280 nm, with theoretical extinction coefficients of 121,590, 118,610, 126,630, and 125,140 M^−1^cm^−1^ for ApePolB1L, ApePolB1S, ApePolB3L, and ApePolB3S, respectively, based on the tryptophan and tyrosine contents (Gasteiger et al., 2003).

### Western blot analysis

The anti-PolB1 and anti-PolB3 antisera were prepared by immunizing rabbits with the recombinant PolB1S and PolB3S proteins, respectively. *A. pernix* K1 was cultivated as described previously (Sako et al., 1996). The cells (0.1 g) were disrupted by sonication in 2 ml buffer, containing 50 mM Tris-HCl, pH 8.0, 0.5 M NaCl, and 1 mM EDTA. The whole cell extracts and the crude recombinant proteins were subjected to SDS-12% PAGE, and the gel was run for a long duration to separate each band. The proteins were transferred onto PVDF membranes (Bio-Rad), which were incubated with the anti-PolB1 and anti-PolB3 antisera. The protein bands were reacted with Immobilon (Millipore), and detected with an LAS-3000mini image analyzer (FUJIFILM).

### Nucleotide incorporation assay

The nucleotide incorporation assay was performed basically as described previously (Uemori et al., 1995). The reactions, containing 25 mM Tris-HCl, (pH 8.6 for PolB1 and pH 8.0 for PolB3), 60 mM NaCl, 10 mM (NH_4_)_2_SO_4_, 0.1% Triton X-100, 0.1 mg/ml BSA, 2 mM MgCl_2_, 2 mM DTT, 0.2 mM each dNTP, 10 μCi/ml [methyl-^3^H]dTTP, 0.2 mg/ml activated salmon sperm DNA, and 20 nM DNA polymerase, were incubated at 70 and 50°C. Afterwards, 10 μl of each reaction mixture was spotted onto DE81 filters (GE Healthcare). The filters were washed with a 5% Na_2_HPO_4_ solution thrice, and were dried. The incorporated radioactivity was measured with a scintillation counter (Aloka). Reactions without enzyme were performed as negative controls.

### Polymerase chain reaction

PCR performances under various conditions were assessed, using λ phage DNA (Takara Bio) as the template and the primers to generate 1-kbp products (5′-dGAGTTCGTGTCCGTACAACTGGCGTAATCATGGCC-3′ and 5′-dCTTTTCAGCCTGGCCCTTTCCTTTACC-3′). The basal PCR solution for ApePolB3 contained 100 mM Tris HCl, pH 8.8, 2 mM MgSO_4_, 50 mM KCl, 10 mM (NH_4_)_2_SO_4_, 0.1 % Triton X-100, 0.1 mg/ml BSA, 0.2 mM dNTPs, and 0.4 μM each primer in a final volume of 50 μl. The basal PCR solution for PfuPolB contained 20 mM Tris HCl, pH 8.8, 2 mM MgSO4, 10 mM KCl, 10 mM (NH_4_)_2_SO_4_, 0.1 % Triton X-100, 0.1 mg/ml BSA, 0.2 mM dNTPs, and 0.4 μM each primer in a final volume of 50 μl. The modified conditions are described in each section. Cycles of PCR, consisting of denaturing at 98°C (10 s), annealing at 55°C (0.5 min), and extension at 72°C (1.5 min), were performed. The products were fractionated by 0.8% agarose gel electrophoresis and stained with ethidium bromide. The PCR fidelity assay was performed using the modified pTV119N plasmid (Takara Bio), containing the *lacZ*α gene. The NcoI- and XhoI-recognition sequences were introduced to the outside edges of the *lacZ*α gene and the original NcoI-recognition site was replaced with an NdeI-recognition site. The resultant plasmid was designated as pTV119NNX. The 483-bp PCR target region was amplified using 0.4 μM each of NF (5′-dCTGGCACGACAGGTTTCCATGGTGG-3′) and XR (5′-dCGTCATCACCGAAACGCTCGAGACG-3′) as the primer set (NcoI and XhoI sites are underlined) and 5 ng of pTV119NNX as the template. Twenty cycles of PCR, consisting of denaturing at 98°C (10 s), annealing at 56°C (20 s), and extension at 72°C (30 s), were performed, using 20 nM ApePolB3 and 20 nM PfuPolB in each basal PCR solution. Each amplified fragment was excised by NcoI and XhoI and inserted into the corresponding sites of pTV119NNX. *E. coli* JM109 cells were transformed with each ligation mixture, and were spread onto LB agar plates containing 50 μg/ml ampicillin, 1 mM IPTG, and 100 μg/ml X-gal. White and pale blue colonies were counted as mutated products while darker blue colonies were considered as intact products. In the amplified region, the 345 bp-fragments corresponded with the expression and function of *lacZ*α. As a background control, excised fragments from pTV119NNX were re-ligated into the corresponding sites of pTV119NNX. White colonies were randomly subjected to colony PCR using the NF/XR primers, to confirm the insertion of the fragment.

### Exonuclease assays

A primed DNA was prepared by annealing 5′-Cy5-labeled 5′-dCGAACTGCCTGGAATCCTGACGACATGTAGCG-3′ and 5′-dTGAGGTGATCGTTCGCTACATGTCGTCAGGATTCCAGGCAGTTCG-3′, in 20 mM Tris–HCl, pH 8.0 and 2 mM MgCl_2_. The ApePolB3 and PfuPolB nuclease reactions were performed with 10 nM substrate in each basal PCR solution, without dNTPs. The reaction mixture was incubated at 70°C and terminated by adding an equal volume of stop solution (98% formamide, 20 mM EDTA, and 0.01% orange G). Aliquots were subjected to 12% PAGE containing 8 M urea in TBE. The gel images were visualized with a Typhoon Trio + (GE Healthcare) image analyzer.

## Results

### Search for the open reading frames of *polB1* and *polB3*

**Figure 1:**
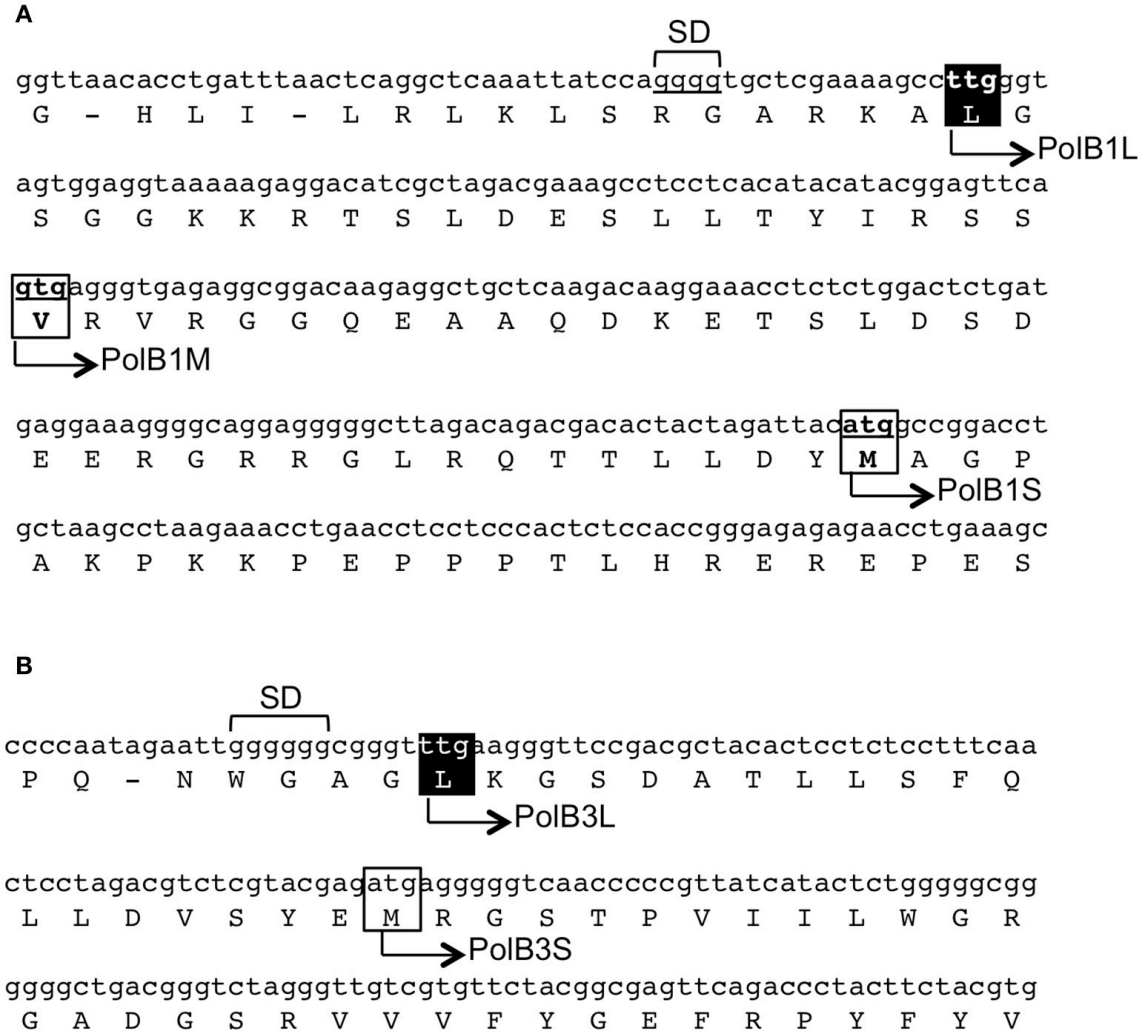
Regions upstream of the initiation sites of the *polB1* **(A)** and *polB3* **(B)** genes from *A. pernix*. The nucleotide sequences of the genome (GenBank, BA000002.3) with the corresponding amino acids are shown. The boxes show the initiation codon candidates. The names of the recombinant proteins in this report are shown on the sequence. The identified initiation sites in this report are indicated in white over a black background. SD indicates the putative Shine–Dalgarno sequences.

In our previous work, two different DNA fragments were amplified from *A. pernix* K1 by PCR, using the degenerate primers based on motif A (SLYPSII) and motif C (VIYGDTD), which are conserved in the family B DNA polymerases, for the forward and reverse sequences, respectively. Approximately 400-bp fragments were amplified from *A. pernix* genomic DNA, and further genomic walking provided the entire structural gene for each *pol*. We deduced the longest in-frames to be the coding regions from their nucleotide sequences, with ATG as the initiation codon. After our analyses, the total genome sequence of *A. pernix* was published and 2,700 ORFs were predicted (Kawarabayasi et al., 1999). Since then, the ORFs were reannotated to 1,871 genes in 2000 (Natale et al., 2000), and furthermore re-evaluated to 1,610 genes in 2004 (Guo et al., 2004). In addition to these re-evaluations, proteome analyses using 2D-gel electrophoresis and mass spectrometry assigned 704 proteins (Yamazaki et al., 2006). One surprising finding was that many proteins (52%) use TTG, rather than ATG (28%) and GTG (20%), as the translational initiation codon (Yamazaki et al., 2006). However, neither of the two DNA polymerases was included in those studies. Each DNA polymerase has multiple candidates for its translational initiation codon, and these structural genes were still unclear even after the annotation accuracy was improved. We searched for the upstream sequences of the *polB1* and *polB3* genes. As shown in Figure 1, we found TTG and GTG, which were in-frame with our original ATG codon for the *polB1* gene. The TTG and GTG extended the ORFs with 58 and 36 amino acids, respectively. For the *polB3* gene, one TTG was found to be in-frame, extending it by 19 upstream amino acids. The amino acid-sequence alignment revealed that the N-terminal portions of the PolB1 homologs had divergent lengths, as well as sequences. The length of the extended PolB1 from *A. pernix* corresponds with those from closely related species (Supplementary Figure 1).

### Identification of the native PolB1 and PolB3 in the *A. pernix* cell extract

**Figure 2:**
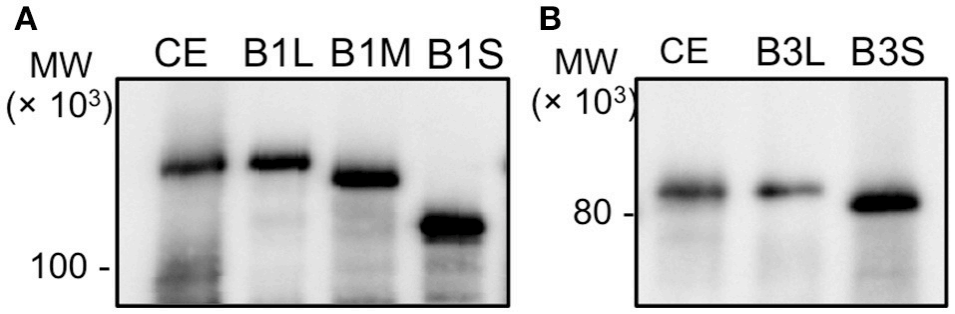
The band mobilities of PolB1 and PolB3 between the cell extracts and the recombinant proteins. Western blotting analyses using anti-PolB1 **(A)** and anti-PolB3 **(B)** are shown. CE indicates crude extracts from 75 μg *A. pernix* cells. B1L, B1M, and B1S indicate the recombinant PolB1 proteins. B3L and B3S indicate the recombinant PolB3 proteins. Pre-stained protein size markers were run in the same gels, and their sizes are indicated on the left.

The recombinant proteins were prepared from *E. coli* cells in our previous study. To identify PolB1 and PolB3 in the *A. pernix* cell extract, anti-PolB1, and anti-PolB3 antibodies were prepared by immunizing rabbits with these purified proteins. The prepared antibodies were used for the western blot analysis to detect the native PolB1 and PolB3 bands from the *A. pernix* cell extract. Three and two ORFs were predicted for PolB1 and PolB3, respectively, as described above. All of these ORFs were cloned and expressed in *E. coli* to prepare each recombinant protein, designated as PolB1L (long), B1M (middle), B1S (short), B3L (long), and B3S (short). To compare the sizes of the bands detected by the western blot analysis, total cell extracts from each recombinant *E. coli* strain were used in parallel with the *A. pernix* cell extract. As shown in Figure 2, the protein bands corresponding to PolB1 and PolB3 were detected in the *A. pernix* cell extract with sizes equivalent to the recombinant PolB1L and PolB3L, respectively. These results suggest that the native PolB1 and PolB3 are produced by the expression of the longest ORFs in *A. pernix* cells.

### Purification of recombinant proteins based on the predicted ORFs

**Figure 3:**
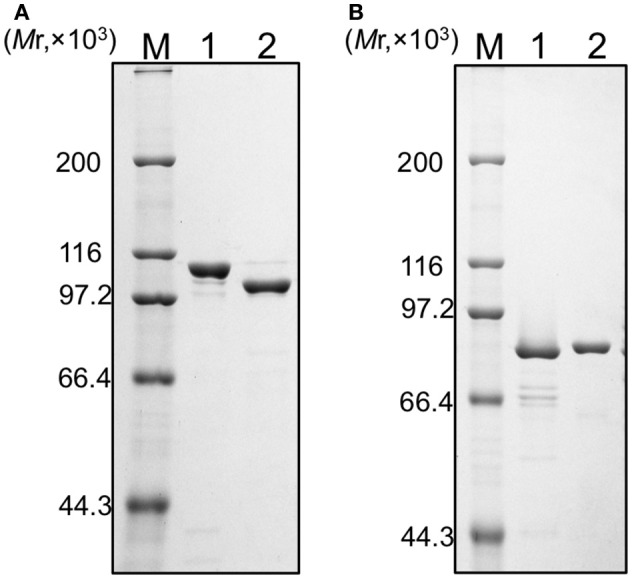
Purification of the recombinant proteins. **(A)** PolB1 proteins (M, protein marker; 1, PolB1L; 2, PolB1S), **(B)** PolB3 proteins (M, protein marker; 1, PolB3S; 2, PolB3L). Purified proteins (1 μg each) were subjected to SDS-10% PAGE followed by Coomassie Brilliant Blue staining. Protein size markers were run in lane M, and their sizes are indicated on the left of the gels.

We tried to purify all of the recombinant proteins for the two DNA polymerases. As shown in Figure 2, all five of the proteins were produced in *E. coli* cells. However, these proteins were mostly insoluble, and especially, PolB1M was not obtained in the soluble fraction at all. Therefore, we proceeded with the purifications of PolB1L, PolB1S, PolB3L, and PolB3S from each soluble fraction, and finally purified these recombinant proteins to homogeneity, as shown in Figure 3.

### Comparison of the specific activities

**Figure 4:**
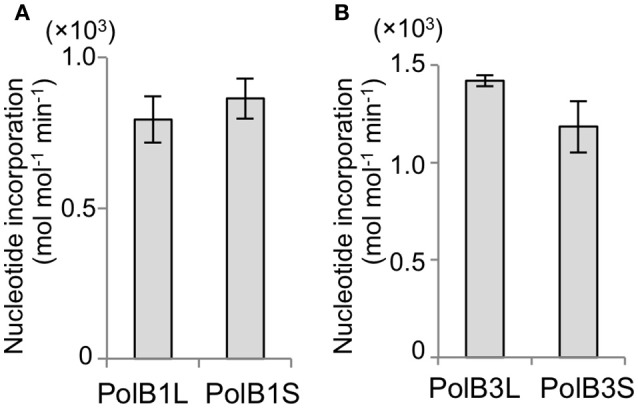
Nucleotide incorporation activities. **(A)** The nucleotide incorporations by 20 nM PolB1 were measured at 50°C for 3 min. Error bars indicate the SEM from three replicates. **(B)** The PolB3 proteins (10 nM) were incubated at 70°C for 3, 6, and 9 min. Error bars indicate the SEM from three replicates.

The activities of the purified recombinant PolB1 and PolB3 proteins were compared using a nucleotide incorporation assay, as described in the Materials and Methods. As shown in Figure 4, the DNA polymerase activity was the same between the long and short proteins for both PolB1 and PolB3. These results suggest that the N-terminal extended portions are not directly involved in the catalytic activities of both PolB1 and PolB3.

### Comparison of the heat stabilities

**Figure 5:**
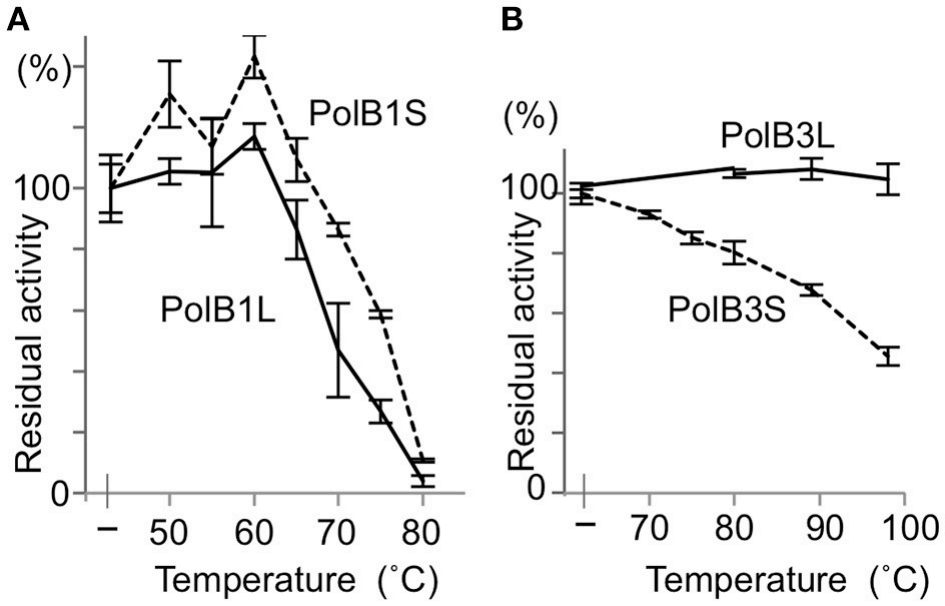
Heat tolerance of DNA polymerases from *A. pernix*. **(A)** The PolB1 proteins were incubated at the indicated temperature for 5 min. **(B)** The PolB3 proteins were incubated at the indicated temperature for 30 min. The residual activities were measured by the nucleotide incorporation assay, using 20 nM PolB1 at 50°C for 2.5 min, and 10 nM PolB3 at 70°C for 5 min. Error bars indicate the SEM from three replicates.

To investigate the functions of the N-terminal extended portions, the heat stability was compared between the long and short proteins for both PolB1 and PolB3. The heat-dependent decrease of the DNA polymerase activity was mostly the same between PolB1L and PolB1S, and both completely lost the activity upon an incubation at 80°C for 30 min (Figure 5A). However, PolB3L showed remarkable thermo-tolerance, and it retained full activity even after an incubation at 100°C for 30 min, in contrast to PolB3S, which gradually lost the activity with increasing temperature (Figure 5B). These results clearly indicated that the N-terminal 19 amino acids are critically important for the stable folding of the PolB3 protein.

### Salt tolerance

**Figure 6:**
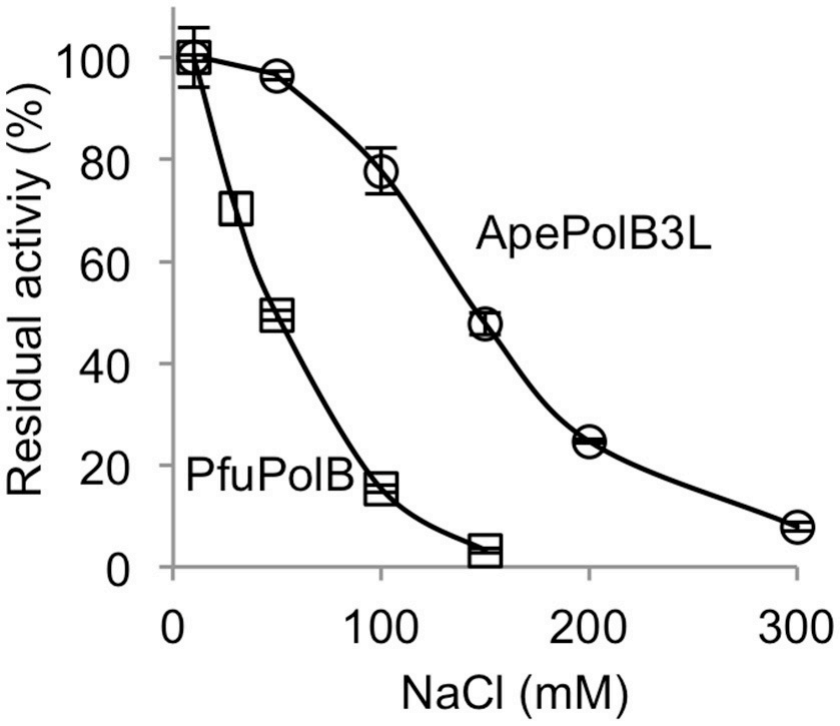
Salt tolerance of DNA polymerases from *A. pernix* and *P. furiosus*. Nucleotide incorporation assays were performed in the presence of various concentrations of NaCl. ApePolB3L and PfuPolB (each 20 nM) were incubated at 70°C for 5 min. Error bars indicate the SEM from three replicates.

The remarkable thermo-tolerance of PolB3L, as described above, suggested that this DNA polymerase may be applicable for PCR. DNA polymerases, including practically used PCR enzymes, are generally sensitive to salt, and the activity decreases with increasing concentrations of NaCl in the reaction mixture *in vitro*. To investigate the salt sensitivity of PolB3L, we compared its DNA polymerase activity with that of *P. furiosus* PolB (a well-known commercial PfuDNA polymerase), which is also highly heat stable, with increasing concentrations of NaCl. As shown in Figure 6, PolB3L showed much higher tolerance to NaCl than PfuPolB.

### PCR performance of PolB3L

**Figure 7:**
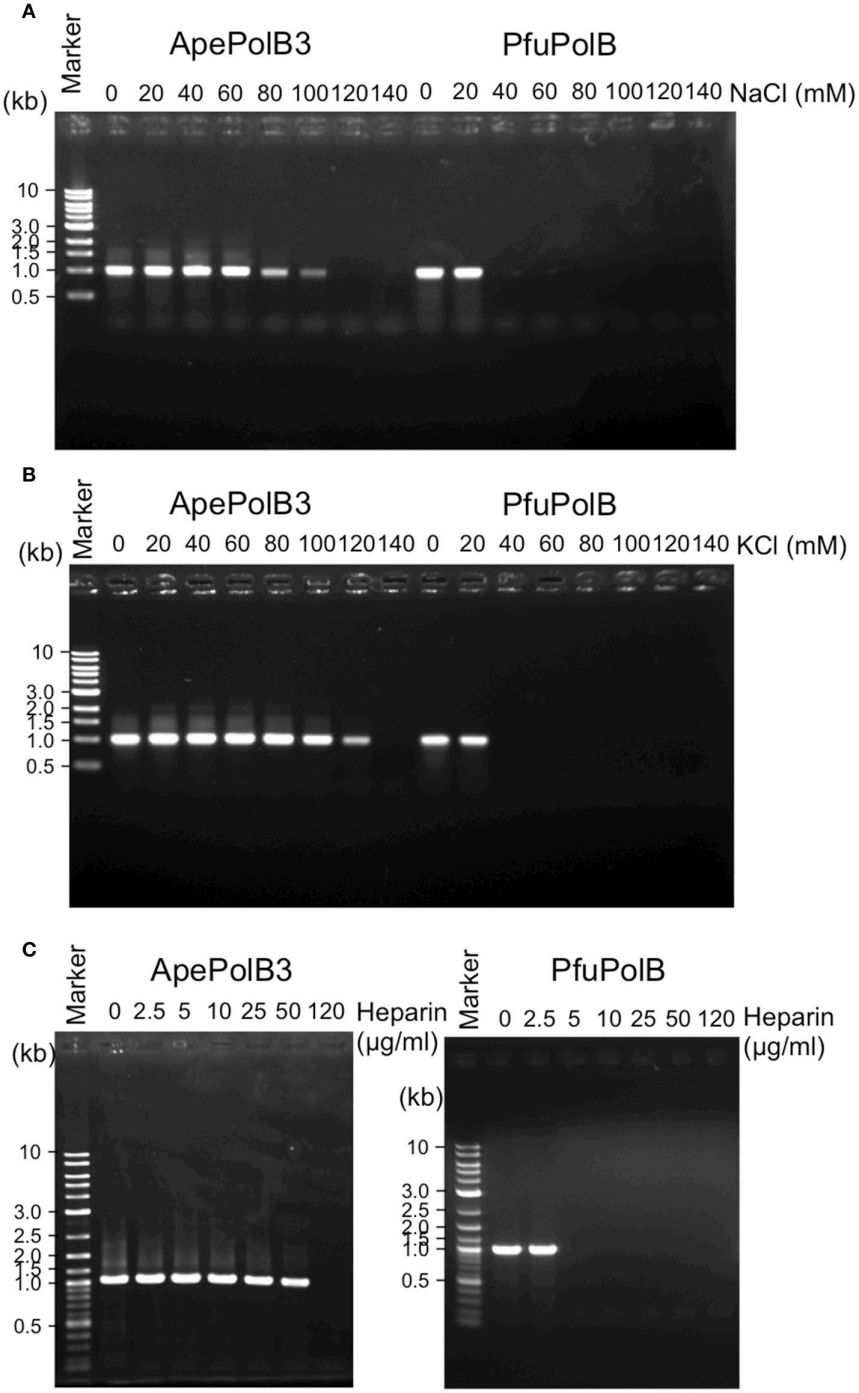
PCR performance of ApePolB3 and PfuPolB. **(A)** and **(B)** Thirty cycles of PCR under each basal condition without any salt including 20 nM DNA polymerase and 0.8 ng λ DNA were performed. The final concentrations of NaCl **(A)** and KCl **(B)** in the reaction solutions are indicated. **(C)** Inhibitory effects of heparin on PCR were assessed with the indicated concentrations of heparin. Thirty cycles of PCR under each basal condition including 30 nM DNA polymerase and 0.8 ng λ DNA were performed.

The above properties suggested that PolB3L may be suitable for PCR using dirty DNA samples. We used PolB3L from *A. pernix* (ApePolB3) for a standard PCR to amplify a 1 kb DNA fragment. Its PCR performances were compared with those of PfuPolB. As shown in Figures 7A,B, ApePolB3 amplified the target DNA in the presence of salt concentrations ranging from 0 to 100 mM NaCl and 0 to 120 mM KCl. In contrast, the amplification by PfuPolB was obviously inhibited from 40 mM of either NaCl or KCl. Furthermore, ApePolB3 was much more tolerant to heparin as compared with PfuPolB (Figure 7C). The apparent mutation frequencies were measured by the amplification of the *lacZ*α gene in the plasmid. The apparent error rates of ApePolB3 and PfuPolB were (2.6 ± 0.5) × 10^−5^ and (1.3 ± 1.6) × 10^−6^, respectively (Table 1).

**Table 1:** Apparent PCR fidelity of ApePolB3 and PfuPolB.

	**Total colonies**	**Mutant colonies**	**Mutant frequency (mf)^a^**	**Error rate^b^**
ApePolB3-1	9,810	966	0.0896	2.0 × 10^−5^
ApePolB3-2	2,422	234	0.0881	2.0 × 10^−5^
ApePolB3-3	2,065	361	0.1488	3.4 × 10^−5^
ApePolB3-4	2,167	280	0.1144	2.6 × 10^−5^
ApePolB3-5	2,923	396	0.1193	2.7 × 10^−5^
PfuPolB-1	2,019	51	0.0246	4.5 × 10^−6^
PfuPolB-2	1,929	23	0.0118	1.4 × 10^−6^
PfuPolB-3	2,376	17	0.0071	2.5 × 10^−7^
PfuPolB-4	2,900	20	0.0068	1.9 × 10^−7^
PfuPolB-5	2,159	16	0.0074	3.1 × 10^−7^
Background	3,599	22	0.0061	

*The data of each DNA polymerase are from five independent assays*.

a *Mutation frequency (mf) is the ratio of the number of mutant colonies vs. the total number of colonies*.

b *Error rate was determined using the equation ER = (mf_sample – mf_background)/(bp × d), where mf is the mutation frequency, bp is the lacZα target size (345 bp), and d is the number of template doublings (12.0) Template doublings were calculated using the equation 2^d^ = (amount of PCR product)/(amount of starting target)*.

### Exonuclease activity of PolB3L

**Figure 8:**
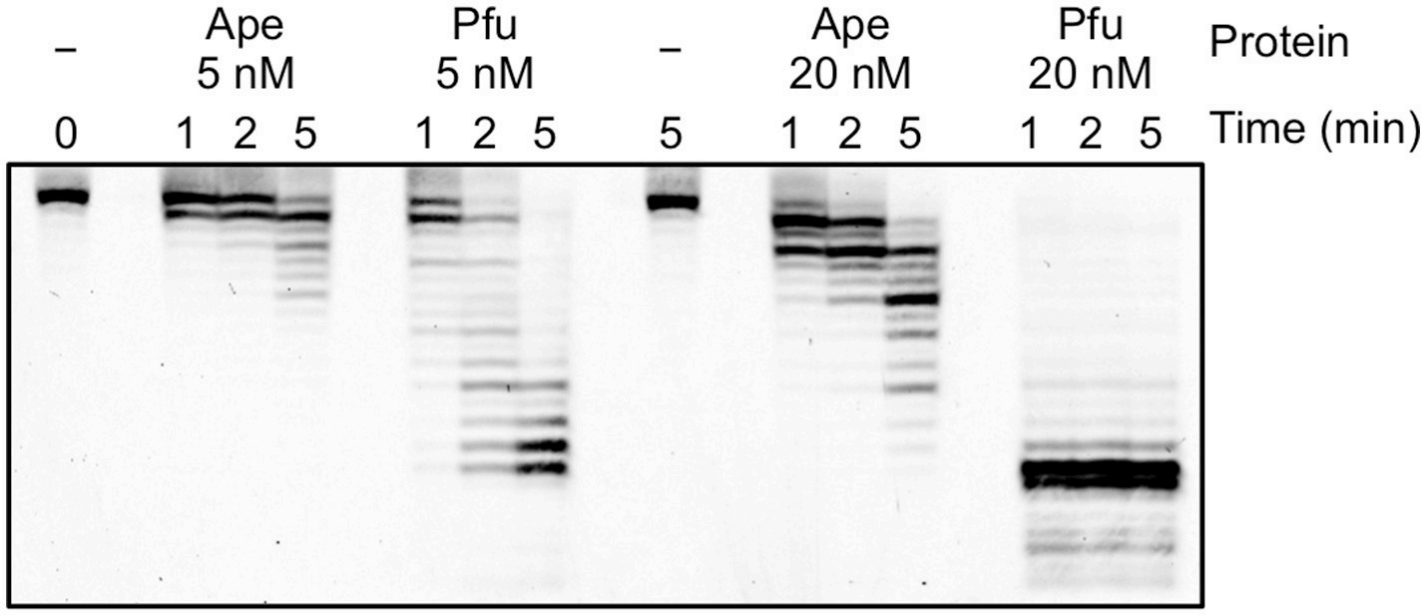
Exonuclease activities of ApePolB3 and PfuPolB. Time course experiments were performed, using 10 nM Cy5-labeled primed DNA. The indicated amounts of ApePolB3 (Ape) and PfuPolB (Pfu) were incubated in the PCR solution without dNTPs at 70°C. For each time point, aliquots were removed from the reaction and quenched by 20 mM EDTA and formamide. The samples were subjected to 12% PAGE, containing 8 M urea, and the degradation products were visualized with a TyphoonTrio imager (GE Healthcare).

To analyze the difference in the apparent error rates between ApePolB3 and PfuPolB in more detail, the 3′-5′ exonuclease activities with ApePolB3 and PfuPolB were compared under each PCR condition, using a 5′-labeled primer and a template substrate. As shown in Figure 8, 20 nM PfuPolB degraded the substrate completely in 1 min. In contrast, 20 nM ApePolB3 only degraded a few nucleotides in 5 min. This result is consistent with the difference in the apparent error rates between the two DNA polymerases. It is currently unknown why the exonuclease activity of ApePolB3 is distinctly weak, as compared with that of PfuPolB, even though the exonuclease motifs are well conserved (Supplementary Figure 2B; Blanco et al., 1991).

## Discussion

*Aeropyrum pernix* K1 is an hyperthermophilic crenarchaeon that was isolated from Kagoshima, Japan, in 1996 (Sako et al., 1996). It aerobically grows at 95°C, and we were interested in how this organism replicates its genomic DNA at such a high temperature. We predicted an ATG, which provided the longest ORF, as the initiation codon to produce recombinant proteins for both DNA polymerases before the genomic era (Cann et al., 1999). Here, we deduced the initiation codons for the translation of these genes more precisely. There is broad variation in both the transcriptional and translational signals in Archaea. In contrast to bacteria, many archaea show wide variations in their consensus Shine–Dalgarno (SD) sequences and frequently use GUG and UUG start codons. The structural genes are generally preceded by SD motifs between positions −15 and −4 in *A. pernix* (Torarinsson et al., 2005). In addition, the significant enrichment of G at position −3 is characteristic for *A. pernix* (Torarinsson et al., 2005). These features are also found in these two structural genes (Figure 1). As described above, *A. pernix* K1 is the first example of an organism, in which TTG is the most predominant translational initiation codon (Yamazaki et al., 2006), as in the two *pol* genes. The precise molecular mechanism of the archaeal translation initiation has not been elucidated, and it is still an interesting question why TTG is most frequently used as the initiation codon in *A. pernix*. The isolation and characterization of the initiation tRNA_i_ will provide some clues for this answer.

What are the functions of the N-terminal portions in PolB1 and PolB3? No difference in the specific activities was detected between PolB1L and PolB1S, and also between PolB3L and PolB3S, indicating that the N-terminal portions are not directly involved in the catalytic function. A remarkable result in this study is that the heat stability of PolB3 was drastically increased with the addition of the N-terminal 19 amino acids (PolB3L), as compared with the protein from ORF from ATG (PolB3S). This difference suggests that the N-terminal 19 amino acids contribute to the stable folding of the PolB3 protein.

It is well known that archaeal family B DNA polymerases have the uracil pocket, which functions to stall synthesis four bases ahead of uracil in the template DNA (this is called the read-ahead function) (Fogg et al., 2002). The amino acids involved in the uracil pocket are highly conserved in these DNA polymerases. The completely conserved Tyr, which is important for the read-ahead function, is present in PolB3L but not in PolB3S (Supplementary Figure 2A), supporting the proposal that PolB3L is the native PolB3 in *A. pernix*.

The lengths and sequences of the N-terminal portions of the PolB1 homologs are especially divergent (Supplementary Figure 1), and their function is currently unknown. A recent report showed that *S. solfataricus* PolB1 is a heterotrimeric enzyme, by the identification of two associated proteins, PBP1 and PBP2 (Yan et al., 2017). The *S. solfataricus* PolB1 complex with these proteins is more heat-stable than PolB1 alone. Their structural analysis showed that PBP1 interacts with the N-terminal and Exonuclease domains, and PBP2 interacts with Thumb domain, located in the C-terminal region. This report described that *A. pernix* also has PBP1 and PBP2 homologs in the genome. However their homologies to these *S. solfataricus* proteins are not obvious, probably because the N-terminal regions of the PolB1 proteins are highly divergent. The heat labile property of *A. pernix* PolB1L may be compensated by PBP1 and PBP2. Furthermore, it is possible that the remarkably long N-terminal region of *A. pernix* PolB1L plays a role in interacting with some other protein factors.

DNA polymerase is one of the most widely used enzymes in genetic engineering. Especially, thermostable DNA polymerases are valuable for applications to PCR-related technologies (Terpe, 2013; Ishino and Ishino, 2014). High heat resistance is required for PCR enzymes, and only the DNA polymerases from extreme thermophiles or hyperthermophiles, but not moderate thermophiles, can be used for this purpose. There are many commercial DNA polymerases from *Thermococcales* in Euryarchaeota, but no crenarchaeal DNA polymerase has been practically used for PCR so far, although several enzymes from *Pyrobaculum* and *Ignicoccus* are reportedly applicable (Kähler and Antranikian, 2000; Ali et al., 2011; Seo et al., 2014). The heat-stability of ApePolB3 is actually sufficient for applications to PCR, and this enzyme is more tolerant to salt and heparin. PolB3S is not applicable for PCR. However, we measured the nucleotide incorporation activity of PolB3S in the presence of 50 mM and 100 mM NaCl, and found the same tolerance as that of PolB3L. Therefore, this property seems to be derived from PolB3S, but not from addition of the N-terminal region. This property is especially advantageous to amplify target DNA fragments from environmental and medical samples with various contaminants. Our simple fidelity test revealed that ApePolB3 is less accurate than PfuPolB, one of the most accurate PCR enzymes. However, the fidelity of DNA polymerases *in vitro* varies with differences in the reaction conditions, and the fidelity of ApePolB3 may increase under different conditions. For example, fidelity of DNA polymerase from *P. abyssi* varied depending on the reaction condition (Dietrich et al., 2002). Furthermore, the more important feature of the PCR enzymes for DNA typing is the efficient amplification of marker genes with precise lengths, and a single base substitution would not have a serious effect on judgment. Actually, the fidelity of *Taq* polymerase, commonly used for this purpose, is much lower than (2.6 ± 0.5) × 10^−5^ shown for PolB3L in this study.

In conclusion, our discovery of PolB3 with native length in *A. pernix*, possessing the remarkable heat-stability and tolerance to salts and heparin, suggests its great potential for the application of this DNA polymerase as a PCR enzyme for diagnostic use from the samples in the medical field and various environments. Furthermore, this study of Family B DNA polymerases from *A pernix* will contribute to understanding of the DNA polymerase active site. In addition, this study may aid in designing of nucleoside inhibitors as therapeutics against viral DNA polymerases and mutants that are resistant to inhibitors.

## Author contributions

YI and SI: Conception and design of study; KD, SI, NI, SN, TY, and HM: Acquisition of data; KD, SI, and YI: Analysis and/or interpretation of data; SI and YI: Preparation of manuscript.

### Conflict of interest statement

The authors declare that the research was conducted in the absence of any commercial or financial relationships that could be construed as a potential conflict of interest.
