# Supplementary material for: Two Family B DNA Polymerases From Aeropyrum pernix, Based on Revised Translational Frames
Source: Front Mol Biosci. 2018 Apr 16;5:37. doi: 10.3389/fmolb.2018.00037 (PMC5911459; doi:10.3389/fmolb.2018.00037)
Supplement: Supplementary file 1 [file DataSheet1.pdf]

Supplementary Material

Two family B DNA polymerases from *Aeropyrum pernix,* based on revised translational frames

Katsuya Daimon, Sonoko Ishino*, Namiko Imai, Sachiyo Nagumo, Takeshi Yamagami Hiroaki Matsukawa, Yoshizumi Ishino

Correspondence: Sonoko Ishino: sonoko@agr.kyushu-u.ac.jp

Supplementary Table S1. Oligonucleotides used for cloning of the ORFs

PolB1L-F	5′-dCGCGCATATGGGTAGTGGAGGTAAAAAGAGGAC-3′
PolB1M-F	5′-dCGCGCATATGAGGGTGAGAGGCGGACAAGAGGC-3′
PolB1S-F	5′-dCGCGCATATGGCCGGACCTGCTAAGCCTAAG-3′
PolB1-R	5′-dGGGGCGGCCGCTCACGAGTCGAAGAGGAGTCTATCTAG-3′
PolB3L-F	5′-dCGCGCATATGAAGGGTTCCGACGCTACACTCC-3′
PolB3S-F	5′-dCGCGCATATGAGGGGGTCAACCCCCGTTATC-3′
PolB3-R	5′-dGGGGCGGCCGCTTATTTCCCCCGCCTCATGAAGTCG-3′

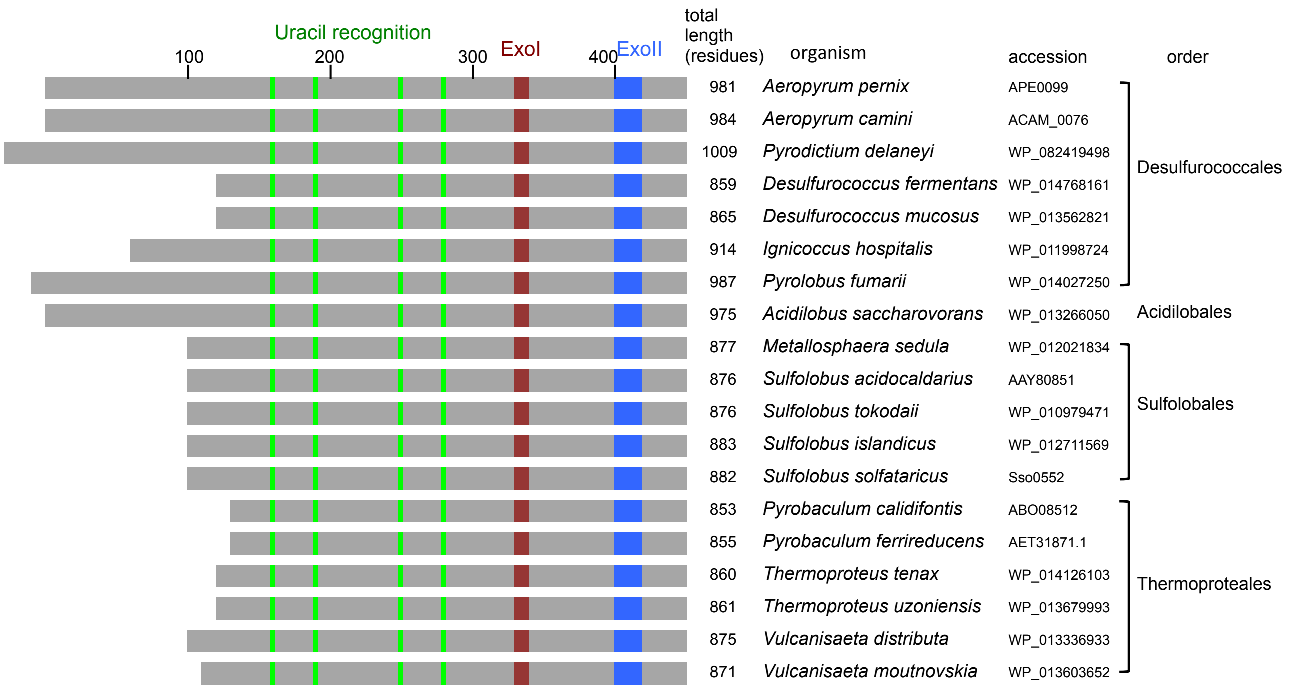

Supplementary Figure 1. A schematic diagram of the alignment of the first 450 residues from ApePolB1L with other PolB1 homologs in Crenarchaeota. The conserved residues and motifs are shown schematically in green (uracil recognizing), red (ExoI) and blue (ExoII).

(A)

```
                                *
Ape   1   MKGSDATLLSFQLLDVSYEMRGSTPVIILWGRGADGSRVVVFYGEFRPYF
Sso   1        MIKDFFILDFSYEIKGNTPLVYIWSVDDEGNSSVVIDNNFRPYF
Poc   1     MTETIEFVLLDSSYEILGKEPVVILWGITLDGKRVVLLDHRFRPYF
Pfu   1            MILDVDYITEEGKPVIRLFK-KENGKFKIEHDRTFRPYI
Tgo   1            MILDTDYITEDGKPVIRIFK-KENGEFKIDYDRNFEPYI

Ape  99   TLVPASVREYREAVRRLGGVRDVLEADIPFALRFIIDFNLYPMRWYVAEV
Sso  87   TSTPTQIKKCREKISELNNIKGIFDADIRYTMRYSLDFDLRPFTWFRAEV
Poc  97   TMIPESVRHYREAVKKIEGVEDSLEADIRFAMRYLIDKRLYPFTVYRIPV
Pfu  87   LEHPQDVPTIREKVREHPAVVDIFEYDIPFAKRYLIDKGLIPME------
Tgo  87   FTHPQDVPAIRDKIKEHPAVVDIYEYDIPFAKRYLIDKGLIPME------
```

(B)

```
Ape  184  LRVMAFDIEVY   242  SIDPDVIVGYNQNRFDWPY   577  VIYGDTDSLF
Sso  166  LRTIGVDFQIY   224  NYDPDIIFVYDSDLLPWKY   537  VILANDLLIF
Poc  181  MRLVAFDIEVY   239  AFDPDIIVGYNSNHFDWPY   576  VIYGDTDSLF
Pfu  135  LKILAFDIETL   200  EKDPDIIVTYNGDSFDFPY   537  VLYIDTDGLY
Tgo  135  LKMLAFDIETL   200  EKDPDVLITYNGDNFAFAY   536  VLYADTDGFF
```

Supplementary Figure 2. Multiple alignment of the conserved residues of DNA polymerase B3. The numbers on the sides indicate the amino acid position relative to the N-terminus. Abbreviated names of organisms and accession numbers are as follows: Ape, *Aeropyrum pernix* (NP_148383.2); Sso, *Sulfolobus solfataricus* P2 (NP_341651.1); Poc, *Pyrodictium occultum* (BAA07580.1); Pfu, *Pyrococcus furiosus* WP_011011325.1; Tgo, *Thermococcus gorgonarius* WP_088885078.1 (A) N-terminal regions of DNA polymerase B3. The previously assumed initiation site is indicated by an asterisk. The conserved residues responsible for uracil-recognition are highlighted in cyan. (B) Conserved residues for exonuclease activity are highlighted in yellow. Ape, Aeropyrum pernix (NP_148383.2); Sso, Sulfolobus solfataricus P2 (NP_341651.1); Poc, Pyrodictium occultum (BAA07580.1); Pfu, Pyrococcus furiosus WP_011011325.1; Tgo, Thermococcus gorgonarius WP_088885078.1
